# Management of bed availability in intensive care in the context of hospitalization by court order*

**DOI:** 10.1590/1518-8345.3420.3271

**Published:** 2020-05-11

**Authors:** Mara Ambrosina de Oliveira Vargas, Elizabeth Peter, Kely Regina da Luz, Edison Luiz Devos Barlem, Carla Aparecida Arena Ventura, Eliane Regina Pereira do Nascimento

**Affiliations:** 1 Universidade Federal de Santa Catarina, Departamento de Enfermagem, Florianópolis, SC, Brazil.; 2 University of Toronto, Lawrence S. Bloomberg Faculty of Nursing, Toronto, ON, Canada.; 3 Universidade do Rio Grande, Escola de Enfermagem, Rio Grande, RS, Brazil.; 4 Universidade de São Paulo, Escola de Enfermagem de Ribeirão Preto, Collaborating Centre OPS/OMS for Nursing Development Research, Ribeirão Preto, SP, Brazil.

**Keywords:** Nursing Care, Intensive Care Units, Ethics, Public Policy, Health Service Management, Health’s Judicialization, Cuidados de Enfermagem, Unidades de Terapia Intensiva, Ética, Política Pública, Gestão dos Serviços de Saúde, Judicialização da Saúde, Atención de Enfermería, Unidades de Cuidados Intensivos, Ética, Política Pública, Gestión de los Servicios de Salud, Judicialización de la Salud

## Abstract

**Objective:**

to identify, from the nurse perspective, situations that interfere with the availability of beds in the intensive care unit in the context of hospitalization by court order.

**Method:**

qualitative exploratory, analytical research carried out with 42 nurses working in adult intensive care. The selection took place by non-probabilistic snowball sampling. Data collected by interview and analyzed using the Discursive Textual Analysis technique.

**Results:**

three categories were analyzed, entitled deficiency of physical structure and human resources; Lack of clear policies and criteria for patient admission and inadequate discharge from the intensive care unit. In situations of hospitalization by court order, there is a change in the criteria for the allocation of intensive care beds, due to the credibility of professionals, threats of medico-legal processes by family members and judicial imposition on institutions and health professionals.

**Conclusion:**

nurses defend the needs of the patients, too, with actions that can positively impact the availability of intensive care beds and adequate care infrastructure.

## Resumo

**Objetivo:**

identificar, a partir da perspectiva de enfermeiros, situações que interferem na disponibilidade de leitos em unidade de terapia intensiva no contexto de internação por ordem judicial.

**Método:**

pesquisa qualitativa exploratória, analítica, realizada com 42 enfermeiros atuantes em terapia intensiva adulto. A seleção aconteceu por amostragem não probabilística, do tipo bola de neve. Dados coletados por entrevista e analisados pela técnica de Análise Textual Discursiva.

**Resultados:**

analisaram-se três categorias, intituladas deficiência de estrutura física e de recursos humanos; falta de políticas e critérios claros para admissão de paciente e alta inadequada da unidade de terapia intensiva. Nas situações de internação por ordem judicial há alteração nos critérios de alocação de leito de terapia intensiva, mediante a credibilidade dos profissionais, ameaças de processos médico-legais por parte de familiares e imposição judicial às instituições e aos profissionais de saúde.

**Conclusão:**

enfermeiros defendem as necessidades dos pacientes, também, com ações que possam impactar positivamente na disponibilidade de leitos de terapia intensiva e de infraestrutura adequada de atendimento.

**Objetivo:**

identificar, desde la perspectiva de los enfermeros, situaciones que interfieren en la disponibilidad de camas en unidad de terapia intensiva en contexto de internación por orden judicial.

**Método:**

investigación cualitativa exploratoria, analítica realizada con 42 enfermeros que prestan servicios en terapia intensiva de adultos. La selección se realizó por muestreo no probabilístico de bola de nieve. Recolección de datos por medio de entrevistas y análisis mediante la Técnica de Análisis de Texto Discursivo.

**Resultados:**

se analizaron 3 categorías a saber: deficiencia de infraestructura y de recursos humanos; falta de políticas y criterios claros para admisión de pacientes y alta inadecuada en unidades de terapia intensiva. En situaciones de internación por orden judicial, se advierten alteraciones respecto de los criterios de asignación de camas de terapia intensiva, mediante la credibilidad de los profesionales, amenazas de interposición de acciones médico-legales por parte de los familiares e imposiciones judiciales a instituciones y a profesionales de la salud.

**Conclusión:**

los enfermeros abogan por las necesidades de los pacientes mediante acciones que puedan impactar de manera positiva en la disponibilidad de lechos de terapia intensiva y de una infraestructura adecuada de atención.

## Introduction

The 1988 Federal Constitution of Brazil guaranteed with citizens the social right to health. As a result, in 1990 the Unified Health System (SUS) was implemented, based on the principles of universality, integrality, equity, decentralization and social participation, with the responsibility of providing public health services for the population^(^ 1 ^-^ 2 ^)^. However, this right to health is often not applied, due to the difficulties faced to guarantee universal and equal access, and from these difficulties emerges the movement for the judicialization of health, especially for access to medicines and health services^(^ 2 ^)^, among which access to an Intensive Care Unit (ICU) bed^(^ 3 ^)^.

People using the public system have access to an average of 0.9 ICU beds *per* 10,000 inhabitants and people with private health insurance have access to 4.14 beds *per* 10,000 inhabitants, the disparity being most pronounced in small towns and cities, in the poorest states of Brazil^(^ 4 ^-^ 5 ^)^. There is a mismatch between the offer in the public health system and the incorporation of new technologies in the SUS and the demand for health care by citizens.

And, both in Brazil^(^ 6 ^-^ 7 ^)^ with in other countries^(^ 8 ^-^ 9 ^)^, the commodification of care is expanded, reflecting the notion that care is considered better distributed by the market, which prioritizes the private health system. It results in a context of State minimization in which health systems, the quality of these services and access to them and the best health technologies are unevenly distributed among the people and groups that make up the social and political-economic organization of society^(^ 10 ^)^.

In the United Kingdom, the number of ICU beds is 0.6/10,000, one of the lowest in Europe^(^ 11 ^)^, even less than the number of SUS beds. However, its physical infrastructure and human resources are superior to those existing in Brazil. In this perspective, in addition to the absolute number of beds in the ICU, there are many reasons for the unavailability of ICU beds in Brazil and other countries, including: limited equipment and drugs; inadequate professional training; high workloads; shortage of professionals; lack of best practices and protocols; aggressive treatment demands; unnecessary ICU hospitalization; relentless treatment inequality in the distribution of resources; lack of availability of regular hospital beds and home care after ICU admissions; competing interests and that influence in the decisions in the screening and inadequate communication with patient families^(^ 1 ^,^ 12 ^-^ 15 ^)^.

In Brazil, in cases of unavailability of a hospital vacancy in the public service, it is the responsibility of the state manager to provide a SUS bed in a private service, guaranteeing the right of access to health services and adequate care. Thus, policies and strategies were introduced that include the Regulation of State Beds and Hospitalization by Judicial Order to assist in the process of finding vacancies. In this circumstance, aware of the gravity of their relative and the need for a bed in another center, the family turns to the Public Prosecutor’s Office or to their lawyer to propose an action to request advance protection. It is an action against the municipality and the State sends it to the State Bed Center^(^ 16 ^)^.

However, it is argued that patients, admitted to the ICU by court order, may not be the ones who most need care in the ICU, which restricts the possibilities for professionals to act according to their ethical and fairness principles. That is, the application of the law, given or ordered by the judiciary, can result in unintended consequences and even harmful to the health of some patients, such as, for example, the transfer of a more severe patient from an ICU to admit a less serious patient^(^ 1 ^)^.

It is an ethical problem that expands, as access to justice and the exercise of rights is restricted to people, through individual actions. In addition, these specific situations limit the possibilities for nurses and other health professionals to defend their patients and work for social justice^(^ 1 ^)^.

The practice of advocacy has been proposed, globally, as a strategy that allows the strengthening of the political, ethical and legal roles of nurses, aiming to ensure their rights and that of the users of the health services in which they work^(^ 17 ^-^ 19 ^)^. Specifically in the ICU, the intensive care nurse has a legal and moral duty to guarantee the quality of care for the seriously ill patient and proactive communication in the decision making of the patient, family, and health team^(^ 20 ^)^.

A Brazilian study^(^ 21 ^)^ coordinates ICU nursing with conceptual aspects of patient advocacy. To this end, when conducting a survey of 451 nurses, it analyzed the actions and factors associated with patient protection by intensive care nurses using the scale Protective Nursing Advocacy Scale, cross-culturally adapted and validated in Brazil^(^ 22 ^)^. And, the study identified that a greater number of nurses understand the defense of the patient as an important part of their work, as well as the factors that can influence the decision to defend their patients, but they are still unaware on the benefits of the advocacy^(^ 22 ^)^.

Patients and family members usually assess care based on the professional’s communication, orientation and positioning skills in the face of the situations experienced. Therefore, it is considered the relevance of the articulation of the theme of the judicialization of health - in this case, hospitalization by court order - to the reflections about the defense for health, human rights and ethics. In this case, the necessary critical improvement of the nurse and his commitment to actions that can positively impact the availability of ICU beds and adequate care infrastructure are highlighted. Thus, the aim of this study was to identify, from the perspective of nurses, situations that interfere with the availability of beds in the ICU in the context of hospitalization by court order.

## Method

Exploratory qualitative analytical research, which belongs to the macroproject, financed by Universal - Ministry of Science, Technology and Innovation and the National Council of Scientific and Technological Development MCTI/CNPq No. 14/2014: Admission by Court Order: The exerciseof patient advocacy by intensive care nurses. Conducted with 42 nurses working in an adult ICU for at least 1 year, regardless of age, sex, working hours, and from public or private institutions, who confirmed having experienced a hospitalization situation by court order. The selection was made by non-probabilistic snowball sampling, and the initial participants were selected by the responsible researcher, according to the inclusion criteria. The participants worked in the ICUs of the capitals of the states of the South region: Curitiba - Paraná (PR), Florianópolis - Santa Catarina (SC) and Porto Alegre - Rio Grande do Sul (RS) and Southeast: Belo Horizonte - Minas Gerais (MG), Rio de Janeiro - Rio de Janeiro (RJ), São Paulo - São Paulo (SP) and Vitória - Espírito Santo (ES), chosen for concentrating 68% of the existing adult ICU beds. The number of participants was determined by data saturation.

The contact was made, first, by telephone or by e-mail and they were asked about the possibility of responding to an interview. After acceptance, the interviews took place in person by the project researchers, on the day, place and time of preference of the participants, with an average duration of 45 minutes. The interviews were recorded with the participants’ permission. Data collection took place between January and December 2016. Data collection instrument contained six questions: 1) Describe any problem or ethical dilemma that you face in view of the need for a vacancy in the indication of hospitalization by court order in the ICU. 2) In the situations experienced by you as a nurse who attends a serious patient needing a bed in the ICU, who is the family member who gets hospitalized by court order? 3) In your point of view, who participates in the process of getting this place in the ICU, through hospitalization by court order? 4) Do you recognize the role of advocacy as an important nursing conduct in this process of getting a place in the ICU? Do you think the nurse’s intervention in this is important? If so, how does this happen or can it happen? 5) Do you know how to obtain a court order? What would you do if you were in the nurse’s place who is there in the emergency room, in the inpatient unit? and 6) What do you think about the consequences of hospitalization by court order in the ICU for the practice of nursing. What is the relationship between hospitalization by court order and the nurse’s responsibility?

For data analysis and interpretation, the Discursive Textual Analysis^(^ 23 ^)^technique was used, following the steps ahead: 1) referred to as the unitarization of texts, resulting from the transcripts of the interviews with intensive care nurses; 2) it comprises the categorization of units of meaning by similarity and approximation, through association with the reference framework used; 3) capturing the new emerging, based on the new combinations of the elements built along the previous steps and that gave rise to the construction of a self-organized process, of reconstruction of new understandings that were communicated and validated in written form.

The study was approved by the Research Ethics Committee, under opinion 863.112. In respect of ethical standards for research involving human beings, the application of the interviews was preceded by the signing of the Free and Informed Consent Form.

## Results

Regarding the characterization of the research participants, it is clear that the nurses’ age ranged from 25 to 54 years old, constituting a young population, with the majority between 25-35 years old. There was a predominance of women with 38 participants, four male nurses. Regarding training time, the average was 12 years. The length of experience in the ICU ranged from 1 to 27 years, with prevalence between 6 and 10 years of experience. And the state capitals had the following distribution of participants: Porto Alegre with 9, São Paulo with 7, Belo Horizonte with 6, and Curitiba, Florianópolis, Rio de Janeiro and Vitória with 5 participants in each of these capitals. In addition, 86% of nurses who participated in the study had specialization in intensive care and 80% worked in public hospitals.

From the data analysis, three categories resulted, entitled: “Deficiency of physical structure and human resources”; “Lack of clear policies and criteria for admission of patient in the ICU” and “Inadequate discharge from the ICU ”.

In the first category, the speeches of the participants highlighted the relationship between hospitalization by court order and the issue of physical structure and materials, equipment and medicines that are deficient or limited: *The professional says: The patient needs better care that is not made available here. Look for your rights, go to court to get an ICU bed with structure, so that it can be monitored with a nursing team and doctor 24 hours a day* (RS6); *Even in large ICUs there are always patients waiting for a place in the emergency room and some are not even in the emergency room and would need a place in the ICU that has the technological and personnel conditions to serve them. The nurse needs to be aware of these social issues and encourage discussions that require more beds from the society* (PR1); *With or without a court order, many patients are waiting for vacancies, including some who do not even have the chance of admission to an ICU and evolve with death*(SC6).

Work overload also impacts the management of ICU beds. It was identified in the nurses’ speeches that, sometimes, they needed to care for critically ill patients in an inappropriate location, adopt specific care with the patient’s family and deal with conflicts between the team, related to the situation of hospitalization by court order: *Patient with judicial referral for surgical intervention, diagnosed with aortic dissection and we had no bed, we had to take him to the operating room and do “pre” monitored in the post-anesthetic recovery room* (RJ2); *Family members are often unassisted, as they are in another city, do not know anyone and are with a sick person. This requires support and support from the ICU team to which the patient will be transferred. The nurse has responsibility, as he will, together with his team, welcome this patient and the family. For example, when talking to the family of a country patient who was hospitalized with H1N1, about where they would be staying, I realized that the financial conditions were poor. I activated the hospital’s social assistance service and a hostel was found for their accommodation* (MG1); *The patient enters the ICU and the bed and box are not prepared. At first everyone asks: Are we going to receive the patient or not? And the patient is waiting and the whole ICU is stopped. This time there was a vacant bed and we forwarded the patient to the bed. One of our problems is the lack of time to prepare. The team thinks that the family will always be there looking for something to bother you, to go to court. I have to say that it is a judicial writ, but it is what the family had [to do] at that moment. I had no problems with the nursing technicians, but I had with the nurses: They already think it’s that family that in any case: I will seek my rights* (RS5).

In the second category called “Lack of clear policies and criteria for admission de patient in the ICU”, the nurses’ speeches expressed the need for clear work guidelines and directives: *The lack of ICU beds is a serious problem in the country. However, I believe that there should be criteria, such as those for assessing patients, before the court order is issued* (RJ4); *The court order arrives “from top to bottom” Judiciary-direction-ICU. On several occasions, the court document requested a place in the ICU, but the intensivists themselves saw no need for it, and an impasse occurred. Even so, the patient was admitted to the ICU by the doctors, leaving the team confused, without knowing the reason for admission to the ICU and, consequently, occupying a bed that could be made available for an emergency. We do not have a responsible sector to clarify these situations that are outside the rule* (SC3).

The issue of alignment between the treatment needs of patients with the priority specialty programs of each hospital was highlighted: *I work in an oncology hospital and a situation that marked, was the admission of a polytrauma patient due to lack of space in the region’s general hospital* (MG3).

The problem of competing interests that influence decisions in the screening of patients for admission to the ICU was also substantiated: *The patient arrived by ICU court order from a private hospital after laparotomy and extubated; but our ICU is highly complex. In this case, there was a position that the patient had no ICU criteria and we suggest that he come and be evaluated in the emergency. And, after the evaluation, he was admitted to the clinical unit and not to the ICU. In another situation, the patient came and returned to the hospital he was in, because in addition to not having a place, he did not need a place in the ICU and the judge agreed. The judge has maximum authority, but if there is a position and a team that points out the criteria for admission to the ICU, he needs to consider* (RS2); *Several times the family forces the arrival of a patient who could stay in a place of medium complexity. And, sometimes it is not possible to assist another patient who really needs the complexity that we offer* (PR2).

Finally, demonstrated in the professionals ‘speeches, the need to deal with conflicts arising from vagueness and inconsistent messages about the patients’ prognosis: *Oncological patient with metastasis, severe pain, and the family requested admission to the ICU for assisted death and the judge’s opinion was positive for the family. The patient needed more comfort, but would it need to be in the ICU?* (SP5).

In the third category referred to as “Inadequate ICU discharge”, unsafe situations on early discharge from ICU patients and the risk of readmission emerged in the participants’ speeches: *Has bad consequences for nursing care; it is unsafe to transfer a patient from the ICU for the other to enter. It is very difficult, because the professional is obliged to do something that he knows is not right at that moment* (RJ3); *The whole situation must be assessed, but it is controversial to have to remove a patient who is in the ICU for another hospitalization. Sometimes, the patient who is discharged early, is not stable and is readmitted to the ICU in the short term* (SC2).

The problem of the difficulty in releasing the patient from the ICU due to the absence of specialized intermediate units in dependent patients was highlighted: *A patient sequeled due to quadriplegia, the family filed an injunction in the court. He remained in the ICU for over a year until he died of recurrent infections* (ES5).

## Discussion

Among the aspects addressed in the “Deficiency of physical structure and human resources” category, there is work overload, linked to the dimensioning of personnel below what is necessary and the precariousness of physical infrastructure and equipment. Therefore, even though hospitalization by court order is a problem in the Brazilian reality^(^ 3 ^-^ 5 ^,^ 12 ^-^ 13 ^,^ 15 ^-^ 16 ^)^, international and national studies address the issue of cost rationalization and access to ICU beds^(^ 4 ^,^ 12 ^,^ 24 ^)^. Rationing is the allocation of health care resources with limited availability.

A multinational^(^ 25 ^)^ that constitutes the largest sample of ICUs in Latin American countries so far analyzed the different structural factors, personnel standards, technological resources and care processes in the ICU. In a comparison between Brazilian and non-Brazilian ICUs, it was shown that Brazil has large hospitals and ICUs and better mechanisms for quality and safety control, but the nurse: patient relationship represents the largest perceived deficit. In this sense, an important study carried out in England, among other aspects, showed that the nurse:patient relationship is an independent determinant of mortality^(^ 26 ^)^.

It is conjectured, here, that the excessive workload and that overlap in the face of the issue of hospitalization by court order is even more complex, since it can impact the difficulty of implementing the multidisciplinary daily care plan and the negative implications of the manifest conflicts among the health team^(^ 27 ^)^. Therefore, there is a need to develop resource allocation strategies in order to optimize assistance in caring for all patients in a fair and responsible manner^(^ 24 ^)^, by means of clear and specific rules and by public policies that even limiting the construction of new ICU vacancies, qualify the existing ICUs.

The deficiency of ICU beds in small cities in Brazil has repercussions on the need to direct patients to large hospitals in capitals and large cities. This is a situation experienced in other countries, for example, in the United States, in which there is a greater probability of increasing the number of ICU beds in teaching hospitals with 500 or more beds, seeking to favor the results of the patient’s treatment. However, the expansion of ICUs in the largest hospitals can negatively reflect on the quality of care in small hospitals, due to the impairment of the ability to care for critically ill patients^(^ 28 ^)^.

Another worrying aspect referred to the effect of the waiting time *per* ICU bed, in situations of hospitalization by court order. In this case, studies^(^ 13 ^,^ 29 ^-^ 30 ^)^ indicate that the refusal of the patient’s access to the ICU or the late admission to the ICU of a patient eligible for the ICU are associated with a higher probability of mortality, disability and additional expenditure of resources due to the longer hospital stay. Still, it can become a serious problem when patients who would need an ICU bed, for example, in the postoperative period of major surgery, are allocated in inappropriate beds and not equipped for the purpose of intensive care 24 hours a day^(^ 28 ^,^ 30 ^)^.

In the category “Lack of clear policies and criteria for patient admission to the ICU”, it could be considered that any analysis of the clear policies and criteria for patient admission to the ICU must be aligned with the ethical objectives of equity, priority of need, or effectiveness, even there is a recognition that the professional would support one or the other of the established criteria. In this sense, the equity of the process can be improved by incorporating some measures: 1) increase publicity for institutional priorities within the hospital and to the community it serves; 2) publication of ICU bed prioritization policies as a reference for professionals and other interested parties; and 3) creating a formal appealing mechanism for conflicts between families and care staff, as a priority program for users and decision makers^(^ 13 ^)^.

Research participants indicated that, in situations of hospitalization by court order, there is a change in the criteria for allocating the ICU bed, through threats of medico-legal processes by family members and judicial imposition on institutions and health professionals. However, adopting criteria is a premise that supports the defense of the patients. Federal Medical Council Resolution No. 2,156/2016 provides parameters for ICU admissions, which are: Diagnosis and patient need; medical services available at the institution; prioritization according to the patient’s condition; availability of beds and, potential benefit for the patient with therapeutic interventions and prognosis^(^ 31 ^)^.

Study^(^ 32 ^)^ signals that English and American guidelines highlight that it may be considered unethical to transfer a patient out of an ICU for the sole purpose of making room for another, as the obligations to ensure the care of patients already hospitalized in an ICU outweigh the obligations to accept new patients. In this sense, there is concern about the imprecision in decision making and the possibility of arbitrariness, even though there was more flexibility for professionals who would apply them in real situations. However, a policy guided by a precision value can give more weight to factors that can be easily measured (quality of life) than factors that cannot (equity and need). Therefore, policy guidelines for resource allocation must be explicit about the ethical values at stake, and how they could be measured^(^ 32 ^)^.

That study^(^ 32 ^)^ corroborates the ethical concern of nurses in this research: the duty to care for the person who is already in the ICU, without risking arbitrariness or inducing wrong or unwise decisions, in the face of unexpected situations of hospitalization by court order. It is critical to examine each particular context and the significance of each possible reason why accuracy can be problematic. The elaboration of guidelines aims to establish limits for decisions, but it should not be intended to prescribe an answer for all possible cases.

Research participants also addressed the issue of *hospitals’ specialty versus versus the need for patient1s treatment.* Studies^(^ 33 ^-^ 35 ^)^ point out that specialized hospitalization can offer qualification for care, and indicate the specialty-screening triad by protocols-clinical experience. Still, bioethicists argue that providing care to all patients in the order in which they arrive represents a rationing of care based on the first-to-care principle, which is inherently flawed because it ignores the relevant differences between people (including the disease) and it is unfair in practice because the richest and best connected can bypass the lines. The importance of doctors and nurses is to be actively involved in reviewing screening policy strategies and in efforts to reduce transfer delays, as well as promoting an understanding of the importance of using an intermediate care unit to minimize the waiting time for the admission of critically ill patients and maximizing the appropriate use of beds for the individual, according to the specialties of each hospital^(^ 34 ^)^.

The nurses ‘discourse regarding the need to deal with conflicts arising from uncertainty and inconsistent messages about the patients’ real prognosis, also reports to the patient’s advocacy, as this professional needs to guarantee the best care available, both for through the monitoring of available treatment, which is sometimes not immediately offered to the patient, as in situations of futility of certain treatments and palliative care. The ICU nurse will not always be able to resolve all the family members’ demands and expectations, but they must be responsible for indicating to the family which means they can use and making sure that the family is having access to these means^(^ 36 ^-^ 38 ^)^.

In the third category, entitled “*Inadequate discharge from the ICU”,* it was considered that the increase in the transfer of patients out of the ICU occurs when the occupation is high, with the consequence of the risk of moving the patients out prematurely. Therefore, even in situations of hospitalization by court order there is an aggravation of the unpredictable, intensivists must be strong defenders of all patients’ needs, regardless of scarcity or expense. And, professionals say that when trying to accommodate new admissions, they realize that their safety standards for transferring patients from the ICU are questionable; the characteristics of the patients they proposed for discharge were less restrictive, which could cause a situation dangerous enough to require an ICU bed^(^ 13 ^)^.

The process of determining the best time to leave the ICU involves a careful assessment of the severity of the disease, as well as the patient’s clinical conditions. Studies have shown that mortality and length of hospital stay are significantly higher in patients readmitted to ICUs after their early discharge^(^ 11 ^)^. Thus, in situations of hospitalization by court order, there is even greater pressure in the early discharge decision, due to the need to release beds for the admission of critically ill patients.

A Brazilian study analyzing the readmission rates of two ICUs (surgical clinic, trauma and neurosurgery), concluded that the readmission of patients in the ICU, during the same hospitalization, resulted in increased morbidity and mortality, length of stay and total costs . Almost half of the patients (46.5%) were readmitted to the surgical clinical ICU within 48 hours of discharge, suggesting early discharge and reaffirming the need and importance of defining criteria for discharge from the ICU^(^ 39 ^)^.

Another study^(^ 40 ^)^, analyzing 33,101 medical requests for 268 public ICU beds regulated in a Brazilian state, found that 55.0% of individuals left the queue before the bed was released due to withdrawal and 20.0% due to death. Among the causes of withdrawal, 47.0% were due to discharge or clinical improvement, 34.0% were transferred by their own means and 9.0% had a diagnosis outside the regulation profile. The authors^(^ 40 ^)^, also, considered that among the 20.0% of deaths in the queue, it would be important to research how many would have been avoided with reduced waiting time or how many patients have already arrived at emergencies out of therapeutic possibilities. In the end, another study of waiting lists in Spain was reported, which showed that timely access, through the management of queue entry with priority levels, has a greater impact than the increase in the supply of beds.

Even though this study was made up of a relatively large group of nursing participants, the restriction of data collection in the South and Southeast regions of Brazil is considered a limitation.

Understanding the reality of ICU nurses in Brazil will not only help inform how to improve decision-making and the development of policies related to the allocation of ICU beds in Brazil. Also, will clarify similar issues faced internationally by health professionals with ICU beds.

Knowing the perspective of nurses working in the ICU regarding the judicialization of access to beds is important. Hence, arising from this discussion, the relevance of nurses and doctors to actively act in the strategies of reviewing screening policies and in efforts to reduce transfer delays, as well as the use of an intermediate care unit to minimize the time of waiting for admission for critically ill patients and optimizing the appropriate use of ICU beds..

## Conclusion

Hospitalization by court order is a problem in the Brazilian reality. Hospitalization by court order is a problem in the Brazilian reality. However, concomitant to this, globally, the issue of cost rationalization and limiting access to ICU beds is growing.

Research participants indicate that in situations of hospitalization by court order there is a change in the ICU bed allocation criteria, through the credibility of professionals, threats of medico-legal processes by family members and judicial imposition on institutions and health professionals.

The excessive workload and the deficiency of ICU beds in small cities in Brazil have repercussions on the need to direct patients to large hospitals in the capitals and large cities. Another worrying aspect refers to the effect of waiting time *per* ICU bed, in situations of hospitalization by court order.

The nurses’ discourse regarding the need to deal with conflicts arising from uncertainty and inconsistent messages about the patients’ real prognosis reports to the patient’s need for advocacy. Ultimately, however, even in cases of hospitalization by court order, ICU nurses must be strong advocates for the needs of all patients, regardless of scarcity or expense.
